# Effortless Precision: A Case Report on the Stamp Technique for Posterior Teeth

**DOI:** 10.7759/cureus.63358

**Published:** 2024-06-28

**Authors:** Khyati Manik, Anuja Ikhar, Aditya Patel, Manoj Chandak, Joyeeta Mahapatra, Jay Bhopatkar, Priyanka R Bhojwani

**Affiliations:** 1 Department of Conservative Dentistry and Endodontics, Sharad Pawar Dental College and Hospital, Datta Meghe Institute of Higher Education and Research, Wardha, IND

**Keywords:** curing, bonding agent, composite restoration, stamp technique, posterior teeth

## Abstract

The stamping technique, a pivotal process, has undergone significant advancements with the integration of composites. Traditionally, direct or indirect restorative cements, e.g., amalgam or composite resin, have been used to restore teeth, often presenting challenges in achieving optimal fit, esthetics, and durability. This process begins with creating an accurate impression of the prepared tooth, which serves as a blueprint for crafting the restoration. In contrast, the stamp technique, also known as the indirect restoration technique, offers several distinct advantages. The stamping technique enables the use of advanced materials that offer superior esthetics and durability. Composite resins used in stamp restorations can be shade-matched to the patient's natural teeth, resulting in seamless integration with the smile. These materials also exhibit excellent strength and wear resistance, ensuring restorations that last longer and are capable of withstanding stresses without fracture.

## Introduction

Nowadays, posterior composite restoration is performed as a routine procedure in dental clinics due to increased concern for esthetics in the anterior and posterior regions [1]. The rapid progress of composite resin restorations is directly linked to the development of minimally invasive restorative procedures that prioritize preserving the natural contour of teeth and using bonding agents in the posterior teeth [1]. The composite restoration still has some limitations, such as time-consuming procedures and the need for superior user abilities to achieve a harmonious alignment between teeth and an ideal cusp-fossa relationship with the teeth in the opposite arch. Other limitations are polymerization shrinkage, microleakage, and discoloration with continued use [2,3].

In addition, a composite restoration takes twice as long to finish and polish as an amalgam restoration [4]. The "stamp technique," a method that involved the use of direct composite resin restorations, was introduced by Waseem Riaz to achieve precise topography of the occlusal surfaces. It has also been documented for use in reconstructing vertical bites in worn dentitions [5].

The stamp technique is highly valued for restorative procedures due to its precision and efficiency. This technique significantly reduces chair time for patients and streamlines the restoration process, enhancing overall treatment efficiency. Additionally, the stamp technique minimizes the risk of errors and material waste, contributing to cost-effectiveness. Its straightforward application also makes it accessible for dental professionals, providing reliable and high-quality results for various restorative needs. This technique requires producing an occlusal index that reflects the anatomy of the occlusal surfaces of the posterior teeth before preparing a cavity [6]. A positive copy of the preoperative anatomy can be obtained by pressing the resulting index against the final increment of the composite before curing. The stamping technique can be used only if the tooth to be treated has intact anatomy. Using the stamp technique, restoring cavitation with hidden caries that are not typically seen is feasible [7]. Due to its ability to replicate the original occlusal anatomy and avoid any deviations from normal shape, this stamping technique is advantageous. The polishing and finishing time of the restoration can be reduced [8].

## Case presentation

**Figure 1:**
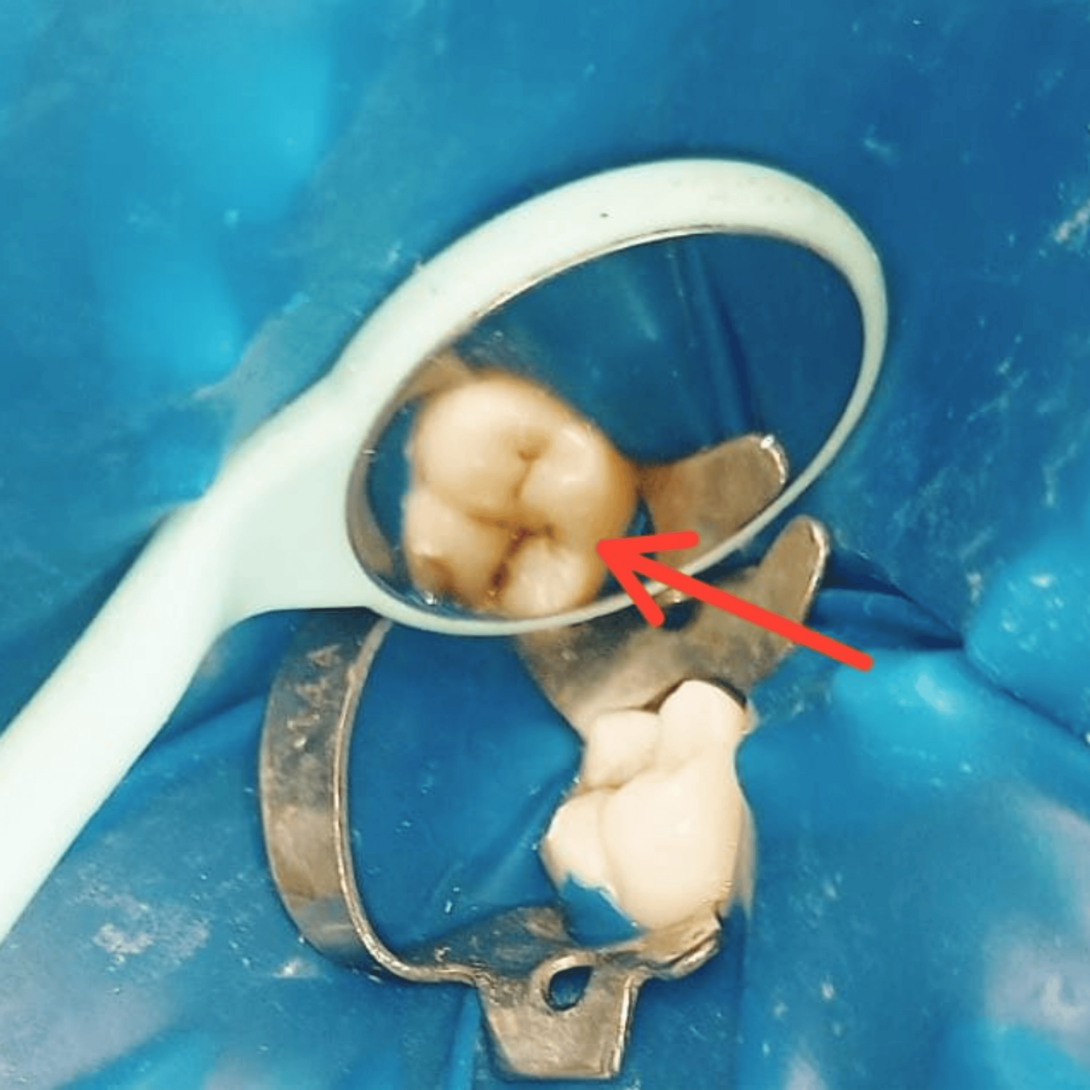
Preoperative photograph (red arrow)

A 20-year-old male patient reported to the Department of Conservative Dentistry and Endodontics at Sharad Pawar Dental College and Hospital, Wardha, with a complaint of black stains on his lower left back tooth region (Figure 1). Past medical history, as well as past dental history, was nonsignificant. Intraoral examination showed pit and fissure caries with the lower right first molar. There was no history of night pain, pus discharge, or swelling. An intraoral periapical radiograph was used to determine the extent of caries. Marginal ridges were intact, and no significant cavitation was seen. Pulp neural sensibility tests, such as electric pulp test and thermal test, have been done. The electric pulp test revealed an early response (reading 7) with the lower right first molar compared to the contralateral lower left first molar tooth (reading 20). Following a hot gutta-percha test, where a 0.06 #15 gutta-percha (Dentsply Sirona, Charlotte, NC) was heated to 65.5°C and placed over the middle third of the mesiobuccal cusp for less than five seconds, the lower right first molar responded with lingering pain upon removal of the stimulus. A diagnosis of chronic reversible pulpitis was made with tooth number 46. Consequently, the decision was made to perform a stamp method using composite resin to reconstruct the decayed area.

Treatment procedure

**Figure 2:**
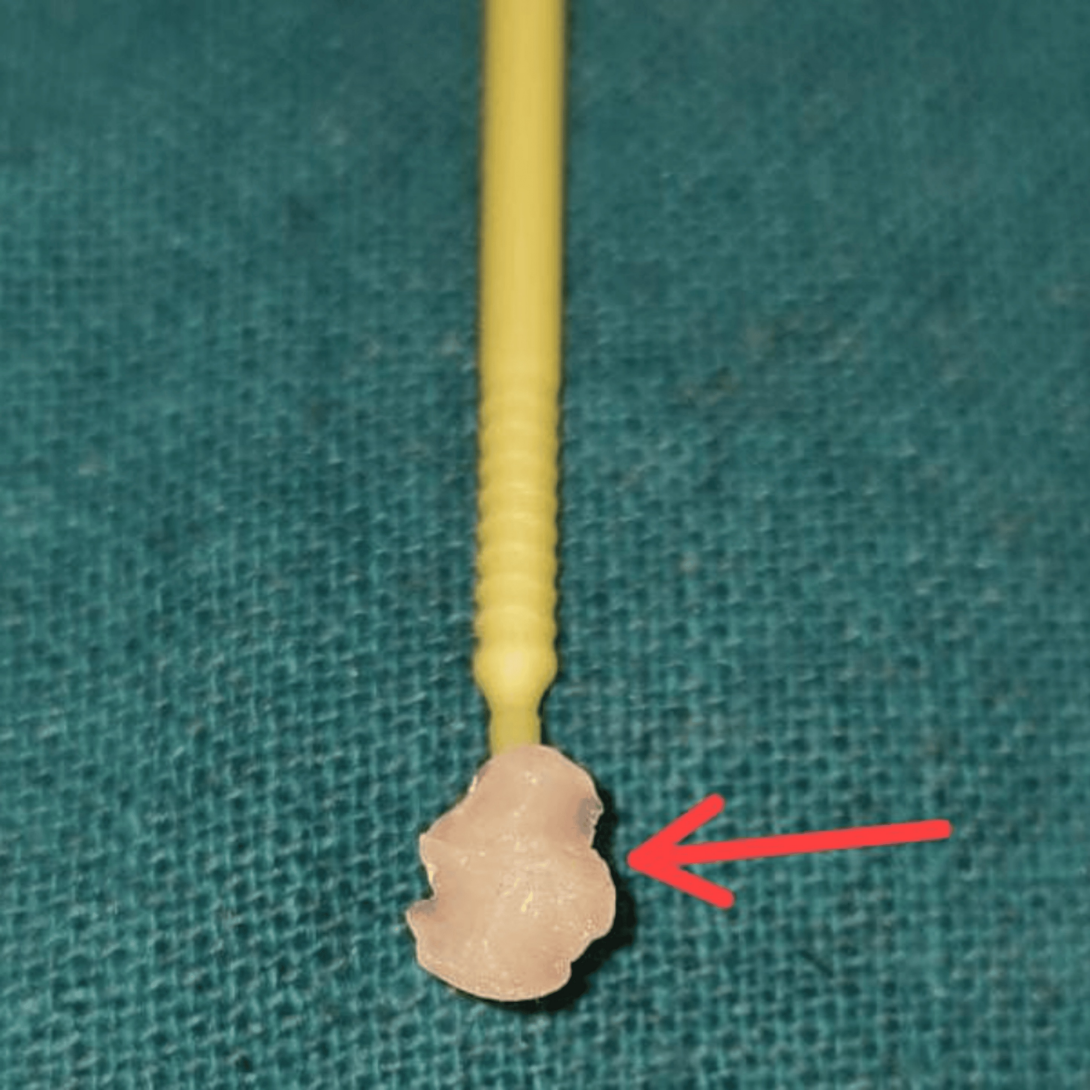
Obtained stamp (red arrow)

Rubber dam (Dental Dam Kit, Global Dental Company, Hoshiarpur, India) isolation was done. Then, with the help of the brush, separating media (Zartex, Zarir & Zaida Industries, Malaysia) was applied on the tooth surface of 46. A stamp or template of the tooth anatomy was created by applying a layer of flowable composite (Tetric N-Flow, Ivoclar Vivadent, Schaan, Liechtenstein) on the occlusal surface of 46. The microbrush tip was trimmed to act as a handle and then carefully inserted into the composite to create the stamp. This was followed by the crucial step of photocuring polymerization (Figure 2).

**Figure 3:**
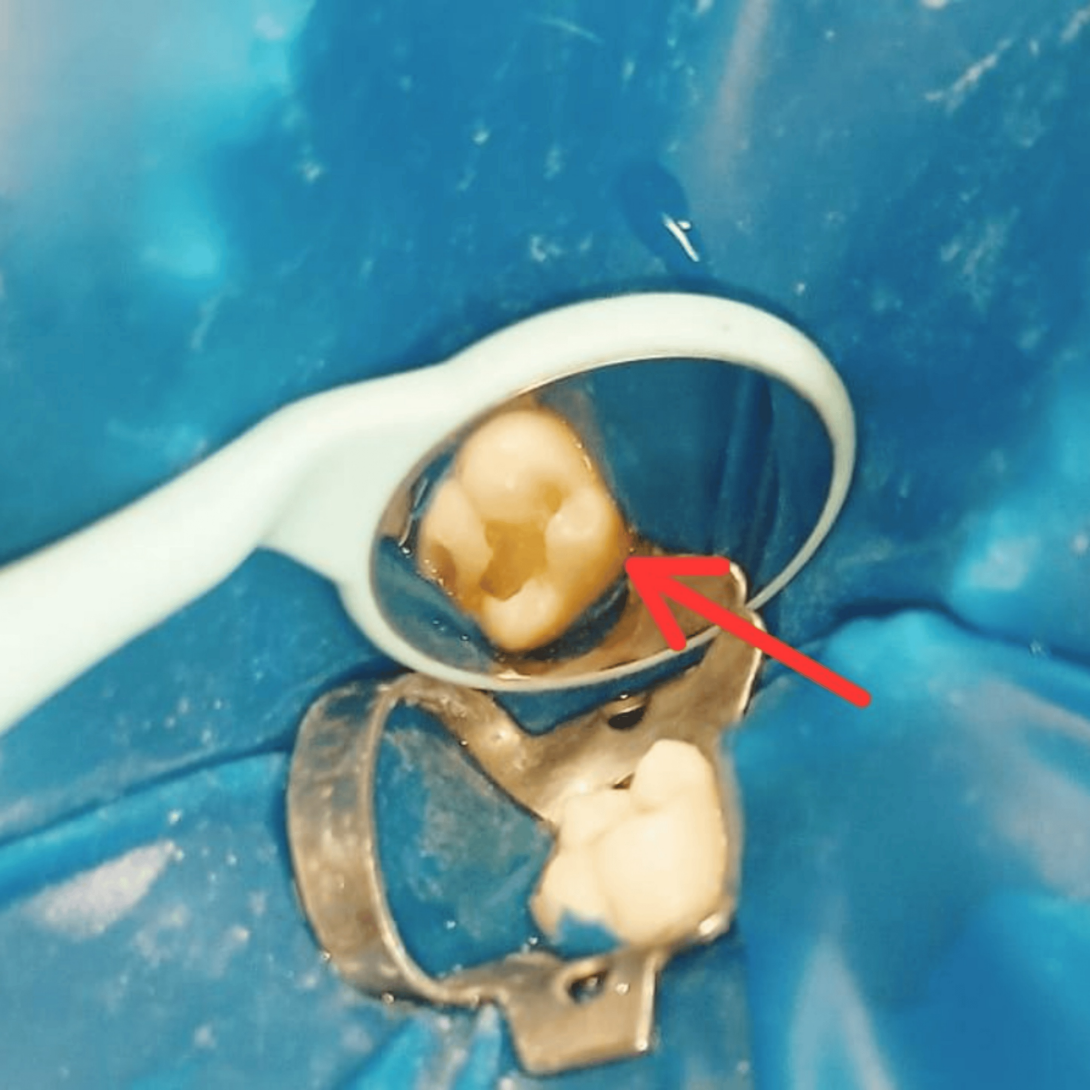
Caries excavation and cavity preparation (red arrow)

The tooth was then prepared for restoration following standard protocols for cavity preparation. Decayed or damaged tooth structure was removed, and a suitable class I cavity shape was created for proper retention and resistance (Figure 3).

**Figure 4:**
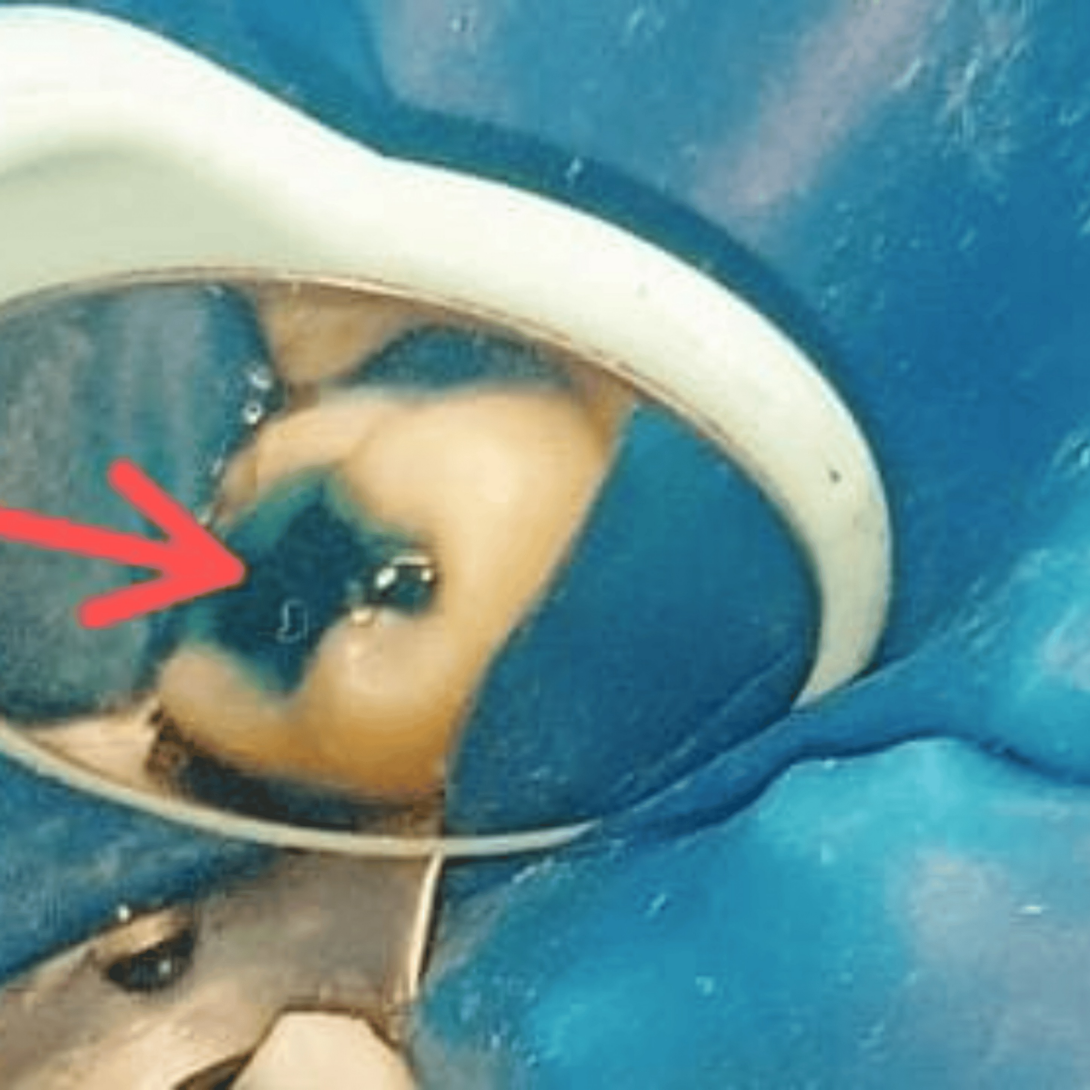
Etching with 37% phosphoric acid (red arrow)

After that, the prepared tooth surfaces were etched for 30 seconds with a dental etchant of 37% orthophosphoric acid (Prime Dental, Ahmedabad, India) to create microretentive surfaces for bonding (Figure 4).

**Figure 5:**
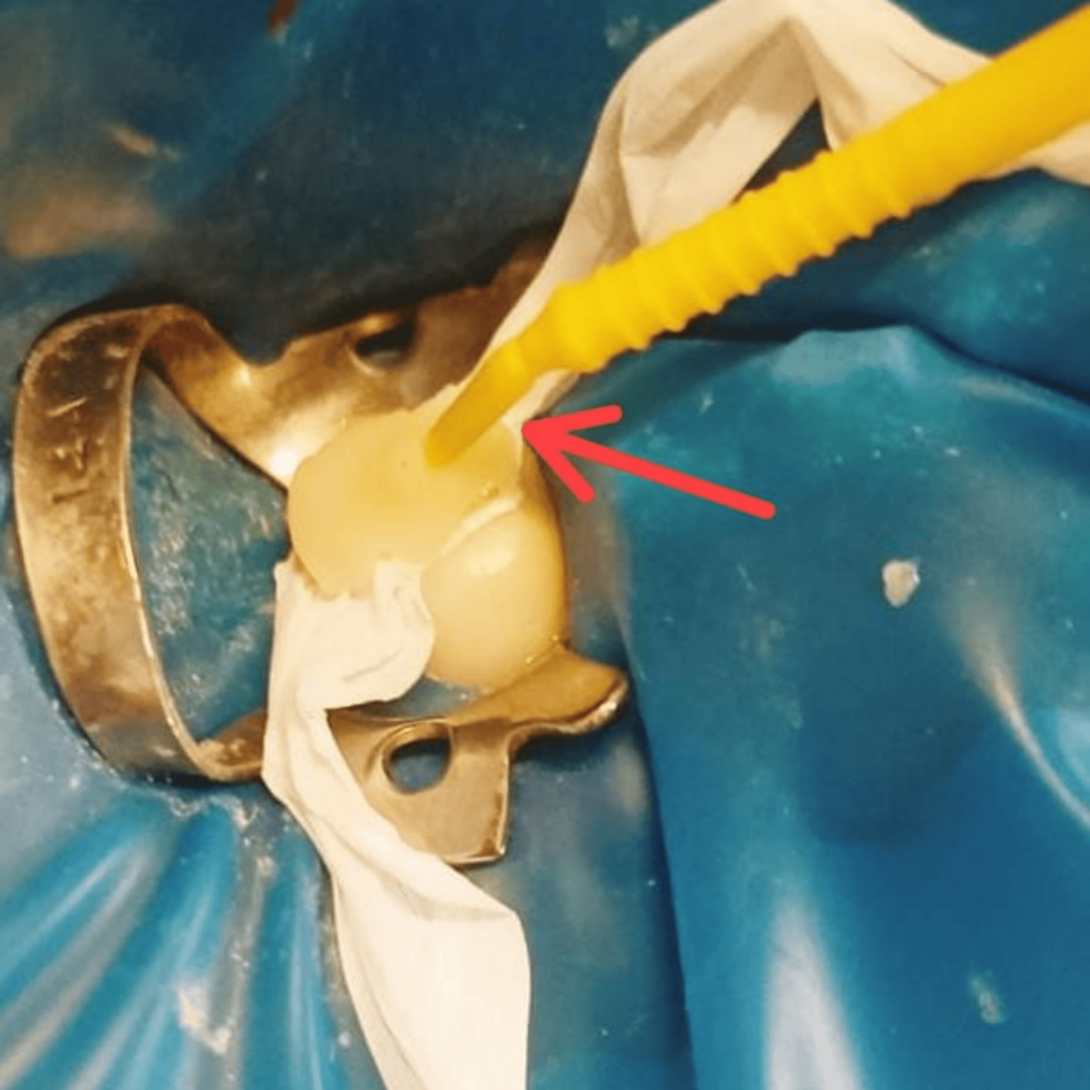
Stamp placement (red arrow)

After rinsing and air-drying with a three-way syringe, the bonding agent (Single Bond Universal, 3M ESPE, St. Paul, MN) was meticulously applied following the manufacturer's precise instructions. Light-cure of the bonding agent was done for 20 seconds to ensure adequate bonding to the tooth structure. The cavity was restored to a depth of 1 mm below the occlusal surface using composite (Spectrum Dentsply Sirona) after 20 seconds of light curing. Before the final curing, a composite layer was carefully applied, followed by the placement of Teflon tape on the occlusal surface. The microbrush occlusal stamp was delicately positioned over the tape and gently pressed with precise pressure (Figure 5).

**Figure 6:**
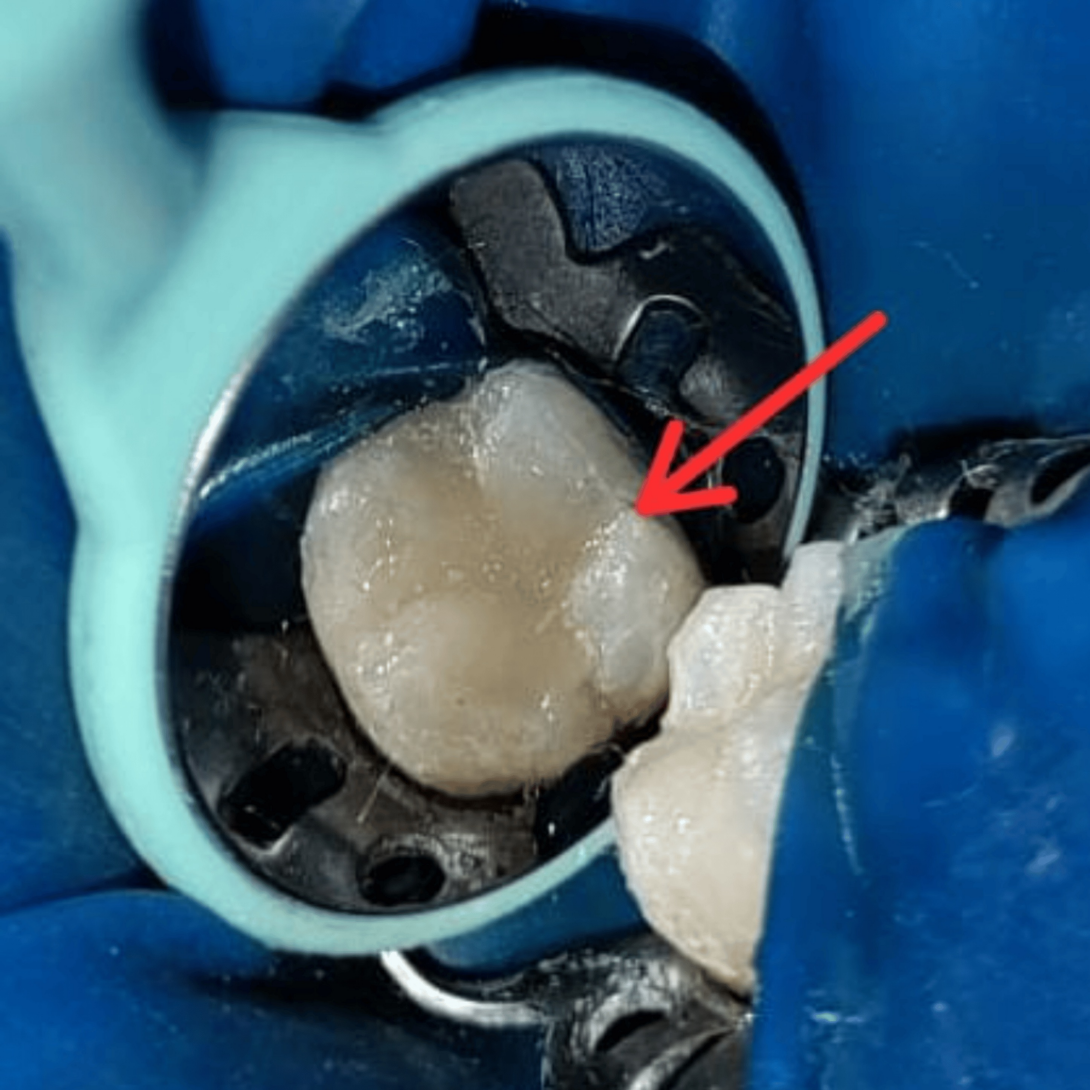
Postoperative photograph (red arrow)

After removing any excess material, the composite was effectively polymerized. Finally, minimal finishing and polishing were expertly carried out using a polishing paper disc (Shofu Super-Snap Mini Kit, Kyoto, Japan) (Figure 6).

Postoperative instructions were given to the patient, including proper oral hygiene practices and dietary restrictions.

## Discussion

Class I cavities can be processed using the stamping technique, which is a straightforward process. No isolating agent is required to apply the flowable composite to the occlusal surface. If the tooth has significant cavities, an isolating agent is recommended. In this condition, the isolating material fills the cavities and does not allow the subsequent flowable compound to flow in. A continuous surface is created for the final restoration. Hence, it is not recommended to use excessive amounts of air spray when applying the isolating agent to the tooth [6].

Stamp technique, like any other technique, has its advantages and disadvantages. One of its significant advantages is the reduced overall time the material is under pressure, leading to a quicker finishing time after recovery. This is extremely beneficial for busy doctors and enhances their reputation among patients [8]. Additionally, the technique significantly reduces the porosity of the final restoration by exerting pressure on the composite, minimizing the formation of microbubbles and the mixing of oxygen during polymerization [9]. However, it is important to consider the downsides, such as the susceptibility to stamp breakage and the cost associated with the flowable composite.

The flowable composite is commonly used because it allows for precise detail reproduction and is readily available. However, it can be costly. One way to reduce expenses is by using expired composites to prepare stamps [10]. Liquid dam material has low viscosity for easy flow but requires a large amount for proper strength due to its high flexibility. Additionally, it is expensive. Transparent acrylic resin is a good material due to its easy handling, low cost, and precision [7]. However, it can create a rough surface under the stamp because it is transparent and can be retained while curing.

Composite resin restorations offer a range of placement methods, encompassing both direct and indirect techniques. Choosing between these methods can be challenging. Single-visit direct posterior restorations offer the benefit of preserving tooth structure and improving its strength, with the possibility of repair [11]. However, there are drawbacks to the direct approach, including proximal and occlusal wear, surface roughness, marginal discoloration, cusp flexure, loss of marginal integrity, secondary caries, postoperative sensitivity, method sensitivity, poor bonding to dentin, and low fracture toughness [12]. On the other hand, the indirect technique reduces the time required to restore multiple teeth in the same quadrant by eliminating contouring and reducing high points, making it beneficial for patients who have difficulty keeping their mouths open for extended periods.

## Conclusions

The stamping technique for composite restorations in posterior teeth is an efficient and effective method for accurately replicating the tooth's natural occlusal anatomy. This technique involves creating a preoperative impression of the tooth's occlusal surface, which is then used to shape the composite material after cavity preparation. The advantages include precise anatomical reproduction, time savings, and reduced need for postcuring adjustments. It also ensures a better occlusal fit and minimizes polymerization shrinkage. While technique-sensitive and dependent on the tooth's initial condition, the stamp technique ultimately offers a minimally invasive approach that enhances both functional and esthetic outcomes, leading to high patient satisfaction.
